# Correlation of cell surface marker expression with African swine fever virus infection^☆^

**DOI:** 10.1016/j.vetmic.2013.12.001

**Published:** 2014-01-31

**Authors:** Pamela Lithgow, Haru Takamatsu, Dirk Werling, Linda Dixon, Dave Chapman

**Affiliations:** a The Pirbright Institute, Ash Road, Pirbright GU24 0NF, United Kingdom; b Royal Veterinary College, Hawkshead Lane, Hertfordshire AL9 7TA, United Kingdom

**Keywords:** African swine fever virus, Surface markers, CD163, MHCII, CD203a, CD45

## Abstract

The expression of surface markers on African swine fever virus (ASFV) infected cells was evaluated to assess their involvement in infection. Previous findings indicated CD163 expression was correlated with ASFV susceptibility. However, in this study the expression of porcine CD163 on cell lines did not increase the infection rate of these cells indicating other factors are likely to be important in determining susceptibility to infection. On adherent porcine bone marrow (pBM) cells the expression of CD45 was strongly correlated with infection. CD163 and CD203a expression correlated at intermediate levels with infection, indicating cells expressing these markers could become infected but were not preferentially infected by the virus. Most of the cells expressing MHCII were infected, indicating that they may be preferentially infected although expression of MHCII was not essential for infection and a large percentage of the infected cells were MHCII negative. CD16 showed a marked decrease in expression following infection and significantly lower levels of infected cells were shown to express CD16. Altogether these results suggest CD163 may be involved in ASFV infection but it may not be essential; the results also highlight the importance of other cell markers which requiring further investigation.

## 1 Introduction

African swine fever is a haemorrhagic fever of domestic pigs and wild boar which is caused by a large cytoplasmic DNA virus, African swine fever virus (ASFV). Isolates of high, moderate and low virulence cause differing levels of disease with mortality ranging from very low to 100%. Macrophages are regarded as the primary target cells for virus replication since at early times, infection is mainly observed in macrophages *in vivo* and has only been observed in other cells at late stages of disease (Carrascosa et al., 1999; Rodriguez et al., 1996). *In vitro*, different subpopulations of monocytes/macrophages vary in susceptibility to ASFV infection but there is little knowledge of the host and virus factors which influence this. Although low levels of infection are observed in freshly isolated bone marrow cells or blood monocytes (McCullough et al., 1997, 1999; Sanchez-Torres et al., 2003) efficient infection is observed in mature alveolar macrophages and *in vitro* maturation of monocytes to macrophages was demonstrated to increase susceptibility to ASFV infection. This correlates with up-regulation of cell surface CD203a (SWC9), a marker of mature macrophages (McCullough et al., 1999; Sanchez-Torres et al., 2003). However, Blocking experiments with monoclonal antibodies to CD203a did not provide evidence for CD203a as a cellular receptor for the virus (Sanchez-Torres et al., 2003).

The expression of CD163, a marker of mature macrophages, has previously been shown to correlate with ASFV infection (Sanchez-Torres et al., 2003). CD163 is a member of the scavenger receptor cysteine-rich superfamily and is expressed on monocytes at low levels and on tissue macrophages at high levels (Kristiansen et al., 2001; Sanchez et al., 1999; Schaer et al., 2006). Antibodies to CD163 were shown to reduce ASFV virion binding to alveolar macrophages by more than 50% (Sanchez-Torres et al., 2003). Furthermore, separation of blood monocytes into CD163 expressing and non-expressing cells demonstrated that permissiveness to ASFV infection correlated with expression of CD163 (Sanchez-Torres et al., 2003). However, in primary cells, it has been shown that ASFV can enter CD163^−^ cells and only a fraction of CD163^+^ blood monocytes are susceptible to ASFV, indicating that additional factors are important for virus replication (Sanchez-Torres et al., 2003).

Additional markers used in this study include CD172a, MHCII, CD16 and CD14, the detection of these markers allows evaluation of the phenotype of the cells. CD172a is expressed on all cells of myeloid lineage (Chamorro et al., 2005). The expression of MHCII, CD16 or CD14 indicates particular activation of the macrophages as they are involved with antigen presentation, antibody binding and LPS detection respectively (Marquet et al., 2011; Chamorro et al., 2005; Carrasco et al., 2001; Ezquerra et al., 2009).

In this study we investigated the cell markers expressed on infected macrophages to further characterise the phenotype of susceptible cells.

## 2 Methods and materials

### 2.1 Preparation and culture of porcine bone marrow (pBM) cells

Bone marrow derived macrophages were obtained from cells rinsed from femur bones with PBS then overlaid onto 1077 Histopaque (Sigma, USA). A gradient was formed by centrifugation at 400 × *g* for 30 min (25 °C) and buffy coat cells were removed into PBS. Cells were pelleted by centrifugation and washed in PBS then cells were re-suspended in Earles saline plus 10% porcine sera (PS) (Biosera, UK) supplemented with 200 U ml^−1^ penicillin and 200 μg ml^−1^ streptomycin and cultured in plastic flasks or plates at 37 °C with 5% CO_2_. Non-adherent cells were removed after 2 h and cells were cultured for a further 6–7 days.

### 2.2 Continuous cell lines

Vero, and PK15 cells, plus cells received from Pfizer Kalamazoo, USA, PK0809, PK9 (express porcine CD163), NLFK-1, FKD4 (express simian CD163) (Calvert et al., 2007) were grown in DMEM medium supplemented with 10% foetal calf serum and antibiotics.

### 2.3 Virus isolates, titrations and infection

ASFV isolates used for infections were BA71v (tissue culture adapted), Attenuated Uganda (tissue culture adapted), Benin 97/1 (high virulence field isolate) and Virulent Uganda (high virulence field isolate).

Field isolates and tissue culture adapted isolates were propagated on pBM cells and Vero cells respectively then purified from cell supernatants using Optiprep gradients (Zhang et al., 2006). Virus stocks were titrated by limiting dilution either in pBM cells, using haemadsorption to detect field isolates or in Vero cells by immunofluorescence as described below for tissue culture-adapted isolates (Goatley and Dixon, 2011; Malmquist and Hay, 1960; OIE, 2007).

For infections virus inoculum was added to the cells in serum free medium. Cells were incubated at 37 °C for 1–2 h; the diluted virus inoculum was then removed and replaced with growth medium supplemented with 10% serum and antibiotics (Vector Laboratories, USA), and viewed on a confocal microscope, Leica TCSSP2.

### 2.4 Detection of CD163 by reverse transcriptase polymerase chain reaction (RT-PCR)

CD163 was detected by RT-PCR, primers were designed to a region of coding sequence of CD163 that is conserved between human, mouse and pig and a conserved region of GAPDH as a positive control. RT-PCR amplifications were performed in 20 μl reaction volumes, using a Onestep RT-PCR kit (Qiagen) following the manufacturer's guidelines. Reaction mixtures included 0.2 μM μl^−1^ of each primer (CD163 forward GGGGACTGAAAGAAGCTGATGT, reverse CCCTCACACTGGAATTCTTCAGCCC, expected product 436 bp; GAPDH forward GCCATCAATGACCCCTTCATTG, reverse TCTTCTGGGTGGCAGTGATGGC, expected product 465 bp) and a variable amount of template RNA (∼250 ng).

### 2.5 Stable expression of CD163 on PK15 cells

Exogenous porcine CD163 was stably expressed in PK15 cells, by transfection of a plasmid expressing the full length porcine CD163, pIRESneoCD163. Cells were transfected with pIRESneoCD163 (coding region for CD163 in pcDNA3 received from the National Institute, INIA, Spain and cloned into pIRESneo data not shown) using TransIT-LTI (Mirus, USA) according to the manufacturer's recommendations. Following transfection, cells were incubated for 18–24 h at 37 °C and 5% CO_2_. Stable expression of CD163 was maintained under G418 selective pressure. Cells expressing CD163 on the surface were purified by magnetic selection, positive selection of cells was achieved using the MACS MS column (Miltenyi Biotec, UK), to select cells positive for CD163. Cells were incubated with 15 ng ml^−1^ anti CD163 primary mouse mAb (2A10, INIA, Spain (Sanchez-Torres et al., 2003)) for 15 min on ice. The cells were then washed three times using serum free DMEM then incubated with goat-anti-mouse-IgG coated micro beads (Miltenyi Biotec) for up to 1 h on ice. Cells were washed three times in MACS buffer and resuspended in 0.5 ml of MACS buffer and positively selected by passing through a MS positive selection column (Miltenyi Biotec). Selected cells were pelleted at 100 × *g* for 5 min. Cells were then resuspended in cell medium and seeded into T25 flasks or processed for limiting dilution.

### 2.6 Immunofluorescence

Cells were grown on 13 mm coverslips in 24-well plates. Cells at different times-post-infection or mock-infected were fixed for 20 min in 4% paraformaldehyde then permeabilised in Ca^2+^/Mg^2+^ free PBS containing 0.2% Triton X-100 for 5 min at room temperature. After washing and blocking in Ca^2+^/Mg^2+^ free PBS containing 0.2% BSA (PBS/BSA) cells were incubated with primary antibody (VP30/CP204L, C18 IgG_1_ (IAH, UK)) followed by secondary antibody (goat anti mouse IgG1 Alexa-Fluor 568, Invitrogen, USA). The coverslips were mounted with Vectasheild containing DAPI.

### 2.7 Flow cytometric analysis

Cells were analysed for surface markers by flow cytometric analysis using the lysis II program on the FACScalibur^®^ (Becton Dickinson, UK). 2 × 10^5^ cells were fixed in 4% paraformaldehyde, permeabilised in Ca^2+^/Mg^2+^ free PBS containing 1% TW20 for 20 min at room temperature and incubated with 20 μl of primary monoclonal antibodies (VP72, 4H3, IgG_2a_ (Cobbold and Wileman, 1998); CD45, K2521.E4 (IAH UK): CD163 [2A10, INIA, Spain (Sanchez-Torres et al., 2003)]: CD203a [C4, IAH UK]; MHCII [K274.3GB, AbD serotec UK]; CD16 [G7, Becton Dickinson, USA], all IgG_1_) for 10 min at room temperature. They were then washed twice with FACS buffer (FB, PBS, 0.1% (w/v) NaN_3_, 2% (v/v) goat serum, 0.3% (w/v) bovine serum albumin), and incubated with isotype specific goat anti-mouse IgG_2a_ antibody conjugated to Alexa-Fluor 488 and goat anti-mouse IgG_1_ antibody conjugated to Alexa-Fluor 633 for 10 min at room temperature, followed by a further two washes with FB. Cells were fixed in 1% PFA and analysed or stored at 4 °C.

## 3 Results

### 3.1 Evaluation of CD163 involvement in ASFV infection

#### 3.1.1 Expression of CD163 in porcine cells

**Fig. 1:**
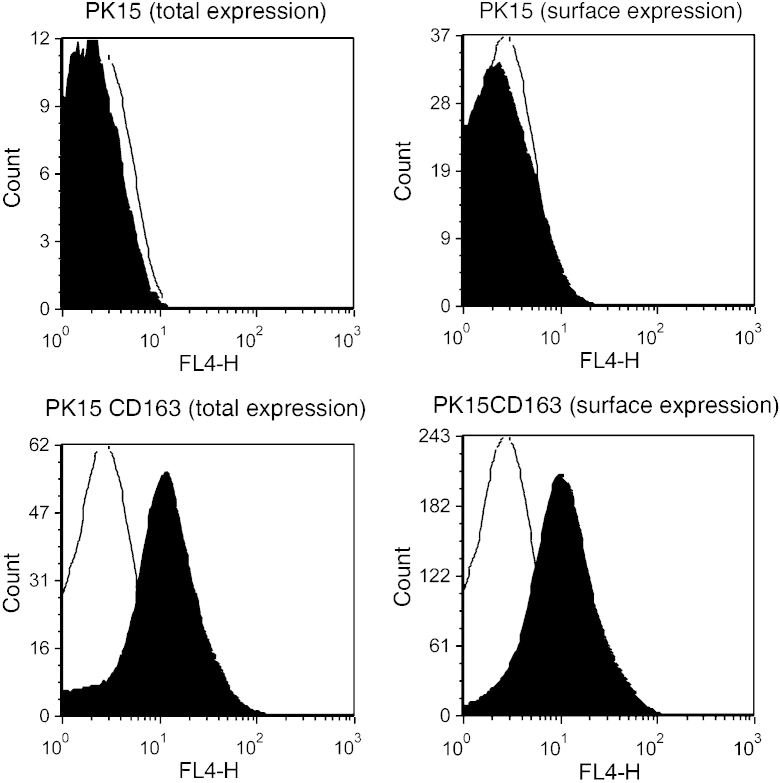
Analysis of CD163 expression on PK15 cells. pBM cells cultured for 6 days, Vero, PK15, and PK15CD163 cells were assessed for the expression of total CD163 in permeabilised cells and on the surface of non-permeabilised cells. Expression was assessed by flow cytometric analysis following staining with mouse anti-porcine CD163 mAb (2A10) and goat anti-mouse Alexa-Fluor 633 conjugated antibody.

Porcine CD163 has previously been suggested as a putative receptor for ASFV. In order to determine if expression of porcine CD163 on the surface of cell lines not expressing CD163 could increase their susceptibility to infection by ASFV we constructed cells lines stably expressing CD163. As a first step several cell lines were assayed for expression of CD163 by RT-PCR. Endogenous CD163 mRNA was detected by RT-PCR in porcine macrophages and Vero cells, but no CD163 mRNA expression was detected in the porcine kidney cell line PK15 (data not shown). Therefore PK15 cells were selected for further studies. Exogenous porcine CD163 (plasmid pIRESneoCD163) was stably expressed in PK15 cells. The expression of CD163 on three clones of PK15 cells (designated PK15CD163) was confirmed by immunofluorescence and FACS (Fig. 1).

#### 3.1.2 Evaluation of ASFV infection of cells express CD163

Experiments were carried out to determine if cell surface expression of CD163 either conferred susceptibility to infection with ASFV field isolates which usually do not infect stable cell lines without adaptation. The effect of cell surface expression of porcine CD163 on replication of tissue-culture adapted ASFV was also evaluated. Cells expressing porcine CD163 (PK15CD163, PK9) or simian CD163 (FKD4) and control parental cells (PK15, PK0809 and NLFK-1) were infected with a tissue-culture adapted ASFV isolate, Attenuated Uganda, which replicates in PK15 cells, or with isolates which do not replicate in PK15 cells (data not shown). These isolates included field isolates Benin 97/1 and Virulent Uganda, which replicate in primary macrophages but not PK15 cells and the Vero cell adapted BA71V isolate. Vero cells and cultured pBM cells were also infected as positive controls for infections with BA71V and field isolates respectively.

PK15, PK15CD163 (3 clones), PK0809, PK9, NLFK-1 FKD4, Vero cells and adherent cultured pBM cells, were infected at a high multiplicity of infection (MOI-10) with ASFV isolates Benin 97/1, Virulent Uganda, BA71V or Attenuated Uganda. At twenty-four hpi adherent cells were fixed and ASFV early viral protein VP30 was visualised by immunofluorescence. Expression of ASFV VP30 is generally observed from about 2 to 4 h post-infection then continually throughout the infection cycle (data not shown). Therefore, detection of cells expressing VP30 indicates that the virus has entered and uncoated and that early virus gene expression has started.

**Fig. 2:**
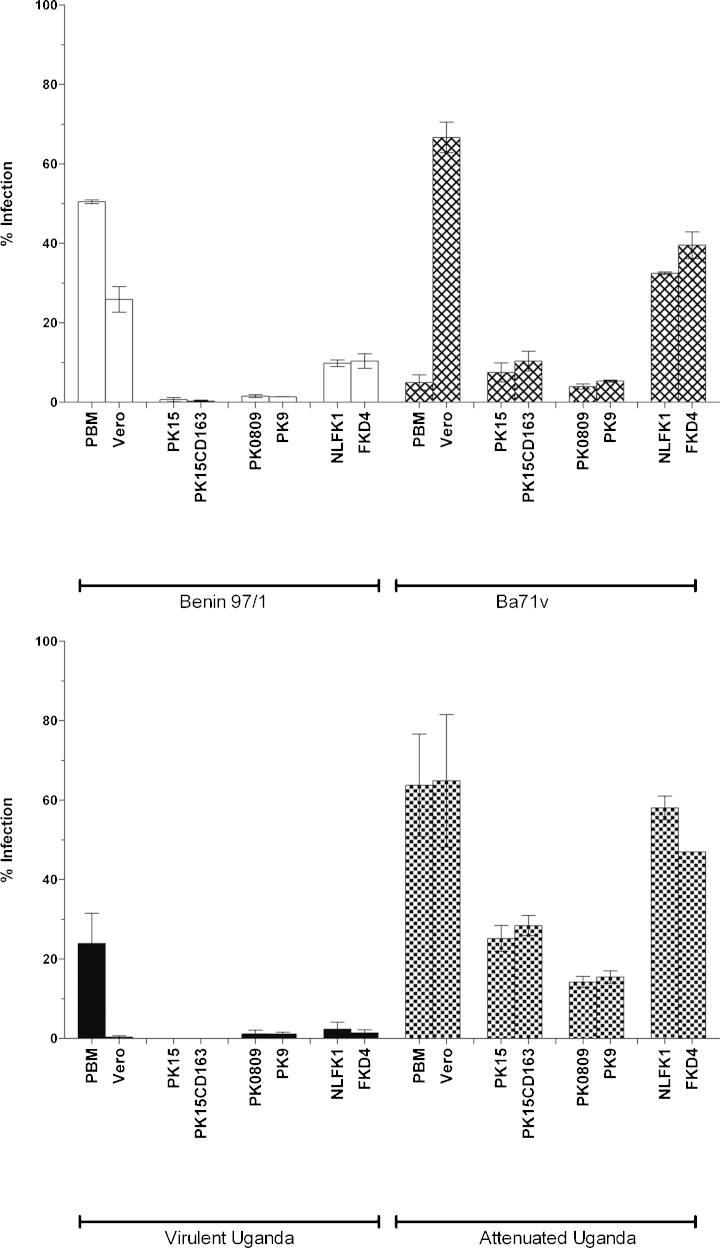
VP30 expression detected in cells with or without CD163 following ASFV infection. pBM cells were cultured for 6 days, Vero, PK15, PK15CD163, PK0809, PK9 (CD163^+^), NLFK1 and FKD4 (CD163^+^) cells (173 ± 6 cells per replicate) were assessed for percentage of cells expressing ASFV VP30 following infection. ASFV isolates Benin 97/1, Virulent Uganda, BA71V or Attenuated Uganda were added at an MOI-10. Twenty-four hpi cells were fixed and permeabilised. Expression of intracellular antigen was estimated by staining with a mouse anti-VP30 mAb (C18) and goat anti mouse Alexa-Fluor 568 conjugated antibody. The virus strain used for infections is indicated under the *x* axis which also shows the cells infected. The *y* axis shows the percentage of infected cells. Bars show the average percentage positive cells and standard error (SE). Results show mean and standard error from 1 to 8 experiments.

Following infection with the Benin 97/1 or Virulent Uganda field isolates, expression of VP30 was detected in 50 ± 0.5% and 24 ± 8% of pBM cells and in 26 ± 3% and 0.3 ± 0.3% of Vero cells respectively (Fig. 2). A very low percentage of cells expressing VP30 were detected following Benin 97/1 or Virulent Uganda infection of all other cells. There was no statistical difference in the percentage of cells which expressed VP30 following infection with the field isolates between the parental cells and cells expressing exogenous CD163.

The percentage of cells expressing VP30 after infection with BA71V or Attenuated Uganda did not differ significantly between parental cell lines and cell lines expressing CD163 (Fig. 2). Thus, expression of exogenous cell surface porcine CD163 did not significantly increase the percentage of cells which could be infected with either field isolates or tissue-culture adapted ASFV isolates.

### 3.2 Evaluation of macrophage surface markers associated with infection

Flow cytometric analysis was used to assess expression of ASFV late viral protein VP72 (B646L) and a range of macrophage surface markers (CD45, CD163, CD203a, MHCII and CD16). The VP72 protein is expressed only at late times post-infection thus its expression demonstrates that virus replication has occurred to this stage. pBM cells were cultured for 6 days in the presence of porcine serum, to allow maturation, before infection with virulent ASFV, Benin 97/1 isolate (Nix et al., 2006) at a high multiplicity of infection (MOI-10). At 24 h post-infection (hpi), adherent pBM cells were processed for flow cytometric analysis with mouse anti VP72 mAb and mouse anti-cell surface marker mAb (CD45, CD163, CD203a, MHCII, CD16).

**Fig. 3:**
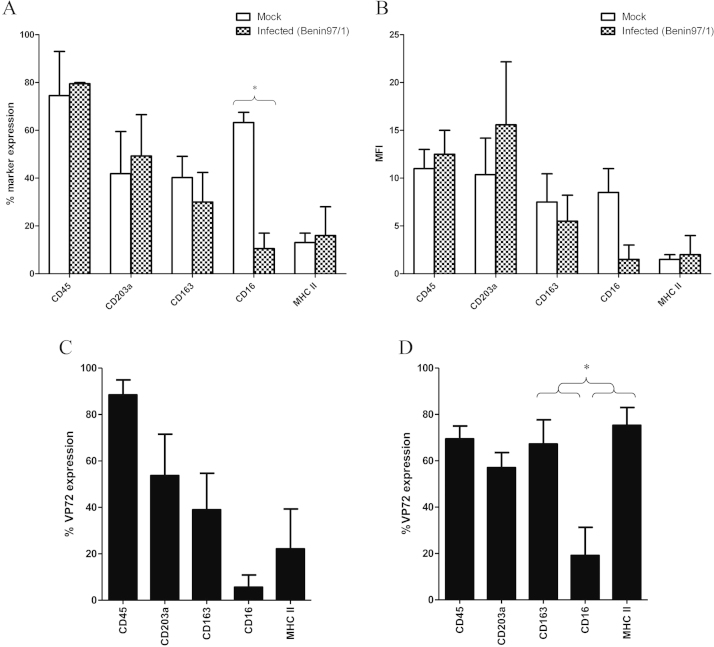
Effect of ASFV infection on cell marker expression of cultured pBM cells. pBM cells were cultured for 6 days then infected for 24 h with ASFV Benin 97/1 (MOI-10). Expression of VP72 (B646L), CD45 (K2521.E4), CD203a (C4), CD163 (2A10), CD16 (G7), and MHCII (K274.3GB) was assessed on adherent pBM cells by flow cytometric analysis. (A) shows marker expression calculated as percent positive events (Overton subtraction method). (B) shows the increase in mean fluorescence intensity (MFI). (C) shows the proportion of each marker expressed within the VP72 positive cell population (marker positive with VP72 positive/total VP72 positive × 100). (D) shows the proportion of cells expressing each marker which expressed VP72 (marker positive with VP72 positive/total marker positive × 100). Results show mean and standard errors from two to four experiments (*p ≤ 0.05).

The percentage of cells expressing the cell surface markers and the intensity of expression, mean fluorescence intensity (MFI, measured using geometric mean) was compared in mock-infected cells and infected cells (Fig. 3). Following ASFV infection, the intensity of expression and percentage of cells expressing CD45, CD203a or MHCII was not significantly different compared to mock-infected pBM cells (percentage: [CD45: infected cells, 80 ± 0.5%; mock-infected cells, 74 ± 18%] [CD203a: infected cells, 49 ± 17%; mock-infected cells, 42 ± 18%] [MHCII: infected cells, 16 ± 12%; mock-infected cells, 13 ± 4%]; MFI: [CD45: infected cells, 13 ± 3; mock-infected cells, 11 ± 2] [CD203a: infected cells, 16 ± 7; mock-infected cells, 10 ± 4] [MHCII: infected cells, 2 ± 2; mock-infected cells, 2 ± 1]). In contrast the proportion of cells expressing, CD163^+^ (infected cells: 30 ± 12%; mock-infected cells: 40 ± 9%) and CD16^+^ (infected cells: 11 ± 7%; mock-infected cells: 63 ± 4%) (*p* = 0.02) were reduced after ASFV infection (Fig. 3A). The intensity of CD16 expression was also reduced following infection (MFI, infected cells: 2 ± 2; mock-infected cells: 9 ± 3) (Fig. 3B).

#### 3.2.1 Evaluation of macrophage marker expression on ASFV infected cells

The proportion of cells expressing each marker expressed within the VP72 positive cell population was calculated (surface marker positive with VP72 positive/total VP72 positive × 100) (Fig. 3C). This allowed evaluation of whether VP72 expression (indicating infection) is restricted to cells expressing any particular marker. A very high proportion of VP72 expressing cells also expressed CD45 (89 ± 6%). The proportion of VP72 expressing cells which also expressed CD163 (39 ± 16%) or CD203a (54 ± 18%) was intermediate. This indicates that cells expressing these markers could be infected, although infection was not dependent on expression of these markers. Within the population of cells expressing VP72, the proportion of MHCII^+^ and CD16^+^ cells was low (MHCII: 22 ± 17%; CD16: 6 ± 5%).

#### 3.2.2 Evaluation of ASFV infection within populations of cells expressing specific markers

To evaluate which cells positive for each marker were infected with ASFV the proportion of cells expressing each marker which were infected was calculated (surface marker positive with VP72 positive/total marker positive × 100) (Fig. 3D). Almost three quarters of the CD45^+^ and MHCII^+^ cells expressed VP72 (CD45: 70 ± 6%; MHCII: 75 ± 8%) and more than half of the CD163^+^ or CD203a^+^ cells expressed VP72 (CD163: 67 ± 10%; CD203a: 57 ± 7%). This indicates that CD45^+^ and MHCII^+^ cells are strongly associated with infection and that CD163^+^ and CD203a^+^ cells can become infected but the expression of these markers is not necessary for infection. Only a fifth of CD16^+^ cells expressed VP72 (19 ± 12%); which is significantly lower than cells expressing CD45 (*p* = 0.07), MHCII (*p* = 0.04) or CD163 (*p* = 0.04).

## 4 Discussion

In these experiments one aim was to determine if cell surface expression of porcine CD163could increase susceptibility of cell lines to infection with ASFV isolates. Previous work had suggested that CD163 expression correlated with susceptibility to ASFV infection and that CD163 may be a receptor for ASFV. However no significant differences were observed when cell lines stably expressing CD163, or the parental cell lines were infected with either virulent field ASFV isolates or tissue-culture adapted ASFV isolates. This indicates that CD163 expression alone is not sufficient to increase ASFV infection in cells that are not susceptible to infection. ASFV infection and replication in these cells may require other molecule(s) such as those necessary to form a receptor complex as well as intracellular factors required for the virus replication cycle. Thus our data does not exclude the possibility that CD163 plays a role in ASFV infection. Previous experiments showed that if cells were treated with antibodies to CD163, ASFV infection was significantly decreased (reduced by 50%). A possible explanation for these findings is that binding of ASFV to CD163 is necessary for some virus infection. However, there are other possibilities such as blocking of virus interaction with other cell surface receptors by the anti-CD163 antibody.

ASFV replicates in cells of monocyte/macrophage lineage and variable infection rates have been reported suggesting that subsets of these cells differing in activation or differentiation state are preferentially infected (McCullough et al., 1999; Sanchez-Torres et al., 2003). Although cell surface expression of CD163 was suggested previously to correlate with ASFV infection (Sanchez-Torres et al., 2003), it was shown that CD163^−^ cells were susceptible to ASFV infection (Sanchez-Torres et al., 2003) and results presented here also show substantial cell numbers in the CD163^−^ population are also susceptible to ASFV infection (60% of ASFV infected cells did not express CD163). No significant change in CD163 expression was observed during the course of infection indicating that the CD163^−^ infected cells did not derive from down-regulation of CD163 after infection. It is important to note that the present study utilised cells which had been cultured for 6 days to produce a mature macrophage phenotype which is more similar to the *in vivo* target for ASFV than the blood monocytes used in the study by Sanchez-Torres et al. (2003). This could in part explain variations in the total level of CD163 cells observed and infected with ASFV.

Our results also showed that intermediate proportions of cells expressing CD45, CD203a were infected with ASFV. Thus, cells expressing these markers could become infected but were not preferentially infected by the virus. Most of the cells expressing MHCII were infected indicating that these cells may be preferentially infected. However, the MHCII^+^ cells represent a small proportion within the total infected cell population and thus expression of MHCII is not essential for infection. In contrast CD16 showed a marked decrease in expression following infection and significantly lower percentages of infected cells were shown to express CD16 compared with the total cell population.

The dramatic decrease in CD16 expression in infected cells and the lack of ASFV infection in CD16^+^ cells indicates that either expression of CD16 is abrogated following ASFV infection and/or the population of CD16^+^ cells are lost from the culture by cell death. If ASFV infection results in down-regulation of CD16 expression this could potentially impact on the function of the cells. Although Sanchez et al. (1999) reported most porcine monocytes and macrophages express CD16, human monocytes/macrophages have subsets of either CD16^+^ or CD16^−^ cells; CD16^−^ monocytes/macrophages were shown to be more immuno-stimulatory and CD16^+^ monocytes/macrophages were shown to be more cytotoxic (Wang et al., 2001; Ziegler-Heitbrock, 2007). Whether such subpopulations of monocytes/macrophages exist in pigs and differ in their function or susceptibility to ASFV infection is not clear and requires further investigation.

The work presented here shows ASFV infection correlates with the expression of specific cell markers not previously identified and suggests these are indicators for cells which have a cellular environment suited to ASFV replication. Further studies are required to continue the investigation into the association of markers and ASFV infection, the experiments presented here evaluated populations of cells produced under a single culture condition. Analysis of macrophages activated in different ways to evaluate changes in marker expression and how this impacts ASFV infection is needed to strengthen the association and possible involvement of the different markers discussed here. Knowledge of the cellular markers associated with ASFV infection will only improve understanding of the virus interaction with host cells and how this impacts on pathogenesis and immune evasion. In addition this could provide information leading to development of improved cell lines for ASFV replication which would have applications in virus diagnosis and vaccine development.
